# The case for ambition: Why countries must move boldly on Near Point-of-Care TB Diagnostics

**DOI:** 10.1371/journal.pgph.0006134

**Published:** 2026-03-23

**Authors:** Sharonann Lynch, Lucy Mupfumi, Collins Otieno, Agrata Sharma, Anafi Mataka

**Affiliations:** 1 Center for Global Health Policy & Politics, Georgetown University, Washington, District of Columbia, United States of America; 2 African Society for Laboratory Medicine (ASLM), Addis Ababa, Ethiopia; PLOS: Public Library of Science, UNITED STATES OF AMERICA

The 2013 World Health Organization (WHO) recommendation of rapid molecular diagnostics as the initial test for tuberculosis (TB) marked a turning point in global TB control, ushering in automated, rapid and more sensitive diagnosis than conventional smear microscopy [1].

More than a decade later, that promise remains partially fulfilled. In 2024 only half of the 8.3 million people with TB were tested with a WHO recommended rapid molecular diagnostic test (WRD), while 2.4 million were missed—neither diagnosed nor notified [2]. The persistent diagnostic gap underscores a broader reality: diagnosis remains the weakest link in the TB care cascade [3]. This gap is attributable in part to significant infrastructure and staffing requirements with current diagnostic tools coupled with high prices [4,5].

Recently, WHO recommended the adoption of newer near point-of-care (nPOC) TB tests. The first in line nPOC TB test is the MTBC Nucleic Acid Test Card from Pluslife – an affordable test that can be performed at primary health care facilities, where people first seek care, using easy-to-collect tongue swab samples [6].

## Why ambition now?

Having the diagnostic tools alone is not enough. History has shown that without ambitious and measurable rollout plans, these innovations can languish for years before reaching the people who need them [5]. This time must be different. The moment demands ambition and timely rollout.

Three factors make this the moment for bold action:

**First, the technology is ready.** The nPOC TB diagnostic pipeline is robust with several products rapidly advancing, including Pluslife MiniDock, Ustar Portnat and LumiraDx TB Tongue Swab Assay [7]. Among them, the Pluslife MiniDock and the Pluslife TB test are now WHO recommended and available for procurement through the Stop TB Partnership’s Global Drug Facility and the Global Fund’s Wambo platform [6,8].

**Second, the financing window is open.** The Global Fund to Fight AIDS, TB, and Malaria Grant Cycle 8 (GC8) represents a critical opportunity to secure funding for nPOC rollout. Countries that develop ambitious, costed roadmaps now and integrate them into National Strategic Plans (NSPs) will be positioned to access this financing. Those that delay may have to wait three years for another chance.

**Third, the human cost of delay is unconscionable.** Every year of slow adoption means millions more people will go undiagnosed, more preventable deaths will occur, and deepening economic hardship will inevitably follow. The 2025 Global TB Report highlights catastrophic cost, often exceeding 20% of their annual income, as a major barrier to care [2]. Moreover, fragmented patient care pathways translate into avoidable repeated visits, lost wages, transport expenses, and prolonged uncertainty [9,10].

## What ambition looks like

Ambitious nPOC rollout must be strategic, planned, and accountable. It requires national roadmaps that set bold targets and create the conditions for success.

### Ambitious targets

**Fig 1:**
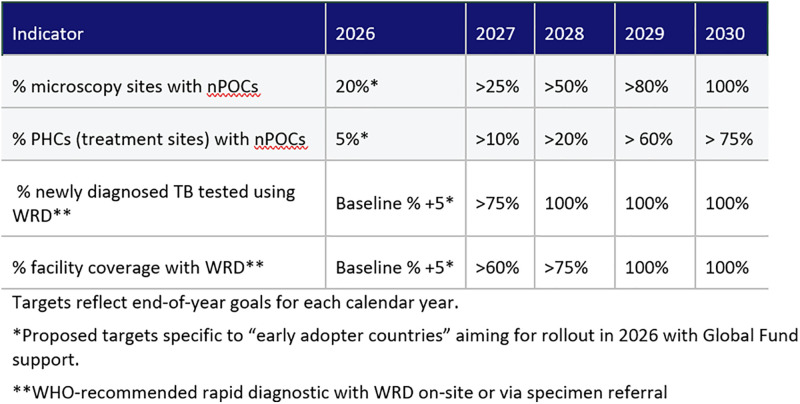
What an ambitious five-year roadmap looks like.

Countries preparing for nPOC implementation should aim for rapid scale-up across their diagnostic networks with an initial focus on replacing smear microscopy as the first test. Microscopy sites represent natural placement locations as the infrastructure and skill set is already in place and they are often located in rural or peri-urban areas where the access gaps are the widest. nPOC devices can also be deployed at busy or underutilized WRD sites as a cost-reduction measure. To reach universal access, countries should prioritize high-volume TB treatment sites, targeting over 20% of primary health care sites to be equipped with nPOC tests by the end of 2028, scaling to over 75% by the end of 2030 (Fig 1). The goal is clear: 100% of people tested for TB using a WHO-recommended rapid diagnostic test, and 100% facility coverage with WRD on-site or via specimen referral.

Global health actors (GHAs) have signaled their commitment to accelerating nPOC rollout: the Global Fund Strategic Initiative, supported by Children’s Investment Fund Foundation (CIFF), along with Unitaid, are funding rapid rollout of nPOC TB diagnostics [11,12]. These coordinated investments are serving a market-shaping function. By aggregating demand and accelerating procurement in “early adopter countries,” GHAs are generating evidence in real world settings, driving volume, increasing predictability for manufacturers, and reducing prices. The cumulative effect is making these diagnostics affordable for the rest of the countries that need access. Therefore, countries have a window to match global momentum with ambitious, time-bound nPOC rollout plans that accelerate equitable access to TB testing.

### Strategic planning, not wishful thinking

Strategic planning should extend beyond procurement and device placement to encompass systematic assessment of service delivery gaps, existing diagnostic capacity, and health system readiness. Effective national roadmaps for the nPOC TB tests must be built on rigorous assessment and deliberate design:

- **Understand the gaps.** Ambition must be targeted at the places where it will save the most lives. Identify where people, such as people living with HIV, rural communities, and the urban poor, are being missed and which regions have the lowest diagnostic coverage.
- **Map the infrastructure.** Assess what diagnostic capacity already exists, including the locations of primary health care sites, microscopy centers, and WRD facilities. nPOC tests should complement existing infrastructure, filling gaps and extending reach rather than duplicating capacity.
- **Optimize placement.** Sites should be prioritized based on TB burden, current access to testing, patient volume, and integration opportunities with HIV and other programs. The goal is to put the right test in the right place for the right populations.
- **Plan for the full system.** nPOC tests require comprehensive training, strong specimen transport networks to ensure linkage to optimal drug susceptibility testing. A positive nPOC result is only the beginning of the care pathway, not the end. (Supporting Information S1 Fig).

### Financial commitment

Ambition requires resources. Countries must develop costed roadmaps that translate targets into budgetary commitments and integrate these into NSPs. Sustained political will and financial commitment will position countries to mobilize external financing.

Investing in diagnostics is both cost-saving and central to strengthening the TB response. Faster diagnosis enables earlier treatment, reduces transmission, and lowers overall health system costs.

The affordability of new nPOC TB tests strengthens the investment case. The Pluslife system costs approximately $335 ($180 for the Thermolyse sample processor and $155 for the MiniDock Ultra reader) compared to approximately $9,420 for the GeneXpert II (one-module with desktop) [8]. The reduced upfront and maintenance costs limit barriers to deployment making decentralization financially feasible. Furthermore, the lower per-test-costs ($3.60 compared to $7.97 for GeneXpert) position nPOCs as a cost-efficient strategy to expand diagnostic access and reduce time to treatment initiation [8]. They could be used for initial screening, while GeneXpert or other WRD are reserved for reflex drug resistance testing when TB is detected. Strategic placement of these molecular diagnostics could translate into savings for TB programs, enabling increased test coverage within existing budgets [13].

### Accountability mechanisms

Effective roadmaps must embed accountability mechanisms, including quarterly reviews to identify and address bottlenecks, public scorecards to track progress, and civil society engagement at every stage to ensure that community voices hold governments accountable to their commitments.

## The stakes could not be higher

nPOC TB tests can transform how TB is detected. Innovative sampling methods such as tongue swabs expand who can be tested, while portable platforms enable decentralized care in remote settings. Rapid results support earlier diagnosis and faster treatment initiation.

These innovations can enable the right test to reach people closer to where they live and seek care if countries choose ambition (Box 1).

### Choose ambition

**Box 1. A call to action**

To national governments: Develop ambitious roadmaps now. Set bold targets. Integrate nPOC diagnostics into National Strategic Plans and GC8 funding requests. Do not wait for perfect conditions—create the conditions for success through committed action.

To donors and global health actors: Reward ambition. Prioritize support for countries that demonstrate political commitment and strategic vision. Create incentives for bold action rather than cautious incrementalism.

To civil society: Demand more. Hold governments to their commitments. Refuse to accept excuses. Be the voice of the millions who remain undiagnosed and untreated.

The window for ambitious action is open. The question is not whether we can achieve universal access to rapid molecular diagnostics—it is whether we will choose to. The lives of millions depend on that choice.

## Supporting information

S1 Fig Structural, operational and financing barriers that have potential to undermine nPOC scale up and must be addressed to match ambition with action. (TIFF)

S1 Text Un Llamado a la Acción. (DOCX)

S2 Text Um apelo à ação. (DOCX)

S3 Text Appel à l’action. (DOCX)
