# Supplementary figures and images for: The case for ambition: Why countries must move boldly on Near Point-of-Care TB Diagnostics
Source: PLOS Glob Public Health. 2026 Mar 23;6(3):e0006134. doi: 10.1371/journal.pgph.0006134 (PMC13008041; doi:10.1371/journal.pgph.0006134)

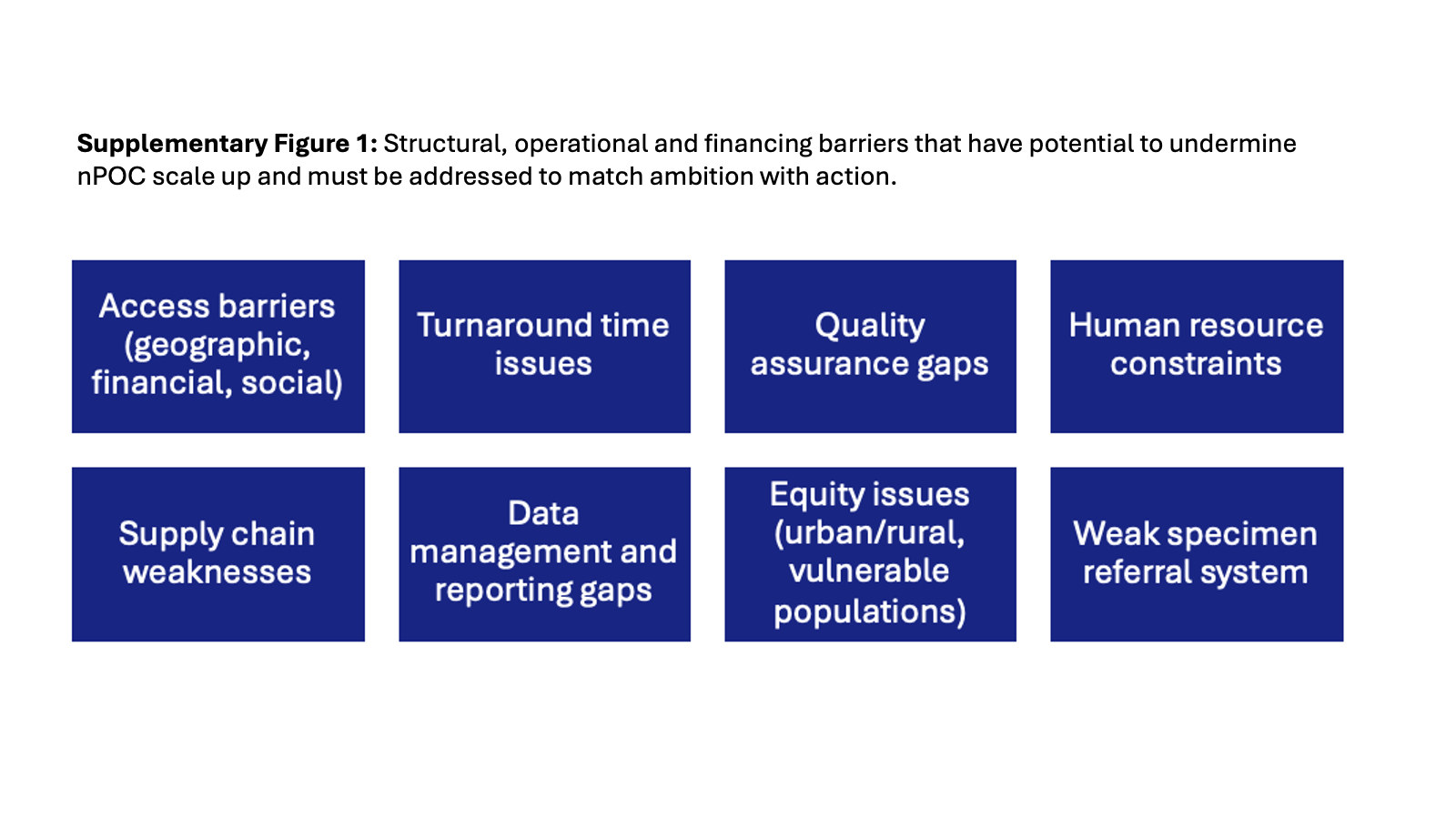

Supplement: S1 Fig — (TIFF) [file pgph.0006134.s001.tiff]
