# Supplementary material for: The case for ambition: Why countries must move boldly on Near Point-of-Care TB Diagnostics
Source: PLOS Glob Public Health. 2026 Mar 23;6(3):e0006134. doi: 10.1371/journal.pgph.0006134 (PMC13008041; doi:10.1371/journal.pgph.0006134)
Supplement: S1 Text — (DOCX) [file pgph.0006134.s002.docx]

**Un Llamado a la Acción**

A los gobiernos nacionales: Desarrollen hojas de ruta ambiciosas ahora. Fijen metas audaces. Integren los diagnósticos cerca del punto de atención (nPOC) en sus Planes Estratégicos Nacionales y en las solicitudes de financiamiento del GC8. No esperen condiciones perfectas: creen las condiciones para el éxito mediante una acción comprometida.

A los donantes y actores de la salud global: Recompensen la ambición. Prioricen el apoyo a los países que demuestran compromiso político y visión estratégica. Generen incentivos para acciones audaces en lugar de un incrementalismo cauteloso.

A la sociedad civil: Exijan más. Exijan a los gobiernos que rindan cuentas de sus compromisos. Rechacen las excusas. Sean la voz de los millones que siguen sin diagnóstico ni tratamiento.

La ventana para una acción ambiciosa está abierta. La pregunta no es si podemos lograr el acceso universal a los diagnósticos moleculares rápidos, sino si elegiremos hacerlo. Las vidas de millones dependen de esa decisión.
