# Supplementary material for: The case for ambition: Why countries must move boldly on Near Point-of-Care TB Diagnostics
Source: PLOS Glob Public Health. 2026 Mar 23;6(3):e0006134. doi: 10.1371/journal.pgph.0006134 (PMC13008041; doi:10.1371/journal.pgph.0006134)
Supplement: S2 Text — (DOCX) [file pgph.0006134.s003.docx]

**Um apelo à ação**

Aos governos nacionais: desenvolvam agora planos de ação ambiciosos. Estabeleçam metas ousadas. Integre os diagnósticos nPOC nos seus planos estratégicos nacionais e pedidos de financiamento GC8. Não esperem pelas condições perfeitas — criem as condições para o sucesso através de ações comprometidas.

Aos doadores e atores globais da saúde: recompensem a ambição. Priorizem o apoio a países que demonstrem compromisso político e visão estratégica. Criem incentivos para ações ousadas, em vez de incrementalismo cauteloso.

À sociedade civil: Exijam mais. Cobrem os compromissos dos governos. Recusem-se a aceitar desculpas. Sejam a voz dos milhões que permanecem sem diagnóstico e sem tratamento.

A janela para ações ambiciosas está aberta. A questão não é se podemos alcançar o acesso universal a diagnósticos moleculares rápidos — é se vamos escolher fazê-lo. A vida de milhões depende dessa escolha.
