# Supplementary material for: The case for ambition: Why countries must move boldly on Near Point-of-Care TB Diagnostics
Source: PLOS Glob Public Health. 2026 Mar 23;6(3):e0006134. doi: 10.1371/journal.pgph.0006134 (PMC13008041; doi:10.1371/journal.pgph.0006134)
Supplement: S3 Text — (DOCX) [file pgph.0006134.s004.docx]

**Appel à l'action**

Aux gouvernements nationaux : élaborez dès maintenant des feuilles de route ambitieuses. Fixez-vous des objectifs audacieux. Intégrez les diagnostics nPOC dans vos plans stratégiques nationaux et vos demandes de financement GC8. N'attendez pas que les conditions soient parfaites, créez les conditions du succès par des actions engagées.

Aux donateurs et aux acteurs mondiaux de la santé : récompensez l'ambition. Donnez la priorité au soutien des pays qui font preuve d'engagement politique et de vision stratégique. Créez des incitations pour des actions audacieuses plutôt que pour un progressisme prudent.

À la société civile : exigez davantage. Veillez à ce que les gouvernements respectent leurs engagements. Refusez d'accepter les excuses. Soyez la voix des millions de personnes qui ne sont toujours pas diagnostiquées et traitées.

La fenêtre pour une action ambitieuse est ouverte. La question n'est pas de savoir si nous pouvons parvenir à un accès universel aux diagnostics moléculaires rapides, mais si nous choisirons de le faire. La vie de millions de personnes dépend de ce choix.
